# To the Medical Profession of New England and New York

**Published:** 1852-01

**Authors:** Worthington Hooker, Henry D. Bulkley, Henry G. Clark

**Affiliations:** Norwich, Conn.; New York; Boston


*To the Medical Profession of New England and New York.*—The undersigned, a Committee of the American Medical Association, on the Epidemics of New England and New York, would invite the attention of the profession within the limits above named to the subject of their investigations.

It is obvious that the value of the report which will be made must depend upon the accuracy and the extent of the information which the committee may be able to gather. And for this information we must look to observers in different portions of the field assigned to us. If the physicians in each district of this field would see that some one of their number report to us what may be called the general facts in regard to the prevalence of epidemics, and then, if individuals would give us the results of their personal experience in practice, a fund of valuable information would be placed in our hands. The points of enquiry to which attention should be directed are so obvious that the committee need not to particularize them. The investigation is intended to cover only the year ending Dec., 1851.

In order that the committee may have time to collate and digest the material which they may receive, they request that all communications be made to them previous to the 1st of March next.

WORTHINGTON HOOKER, Norwich, Conn.
HENRY D. BULKLEY, New York.
Dec. 15, 1851. HENRY G. CLARK, Boston.

